# Scaled-up nutrition education on pulse-cereal complementary food practice in Ethiopia: a cluster-randomized trial

**DOI:** 10.1186/s12889-020-09262-8

**Published:** 2020-09-22

**Authors:** Getenesh Berhanu Teshome, Susan J. Whiting, Timothy J. Green, Demmelash Mulualem, Carol J. Henry

**Affiliations:** 1 School of Nutrition, Food Science and Technology, Hawassa University, Hawassa, Ethiopia; 2 College of Pharmacy and Nutrition, University of Saskatchewan, Saskatoon, Canada; 3 SAHMRI, Australia, Adelaide, Australia

**Keywords:** Complementary food, Dietary diversity, Germination, Health extension workers, Nutrition education, Pulses, Scale-up

## Abstract

**Background:**

Improving children’s weight status through nutrition education (NE) for mothers about using pulses in complementary feeding has been demonstrated in pilot studies, but no effect on stunting was reported. The aim of the study was to assess the impact of a 9-month pulse-nutrition education program on improving mothers’ knowledge, attitude, and practices (KAP) towards pulses, as well as its effect on children’s diet diversity, and nutritional status. The NE was delivered by Health Extension Workers (HEWs).

**Methods:**

A cluster randomized study was employed for the community-based interventional study*.* Twelve randomly selected villages in Sidama Zone, Southern Ethiopia were included in the study. A total of 772 mother-child pairs involved in the study; where 386 mother-child pairs in the intervention group received additional messages about pulse-cereal complementary food, and 386 pairs (the control) received only routine health education for 9 months. A survey on mothers’ KAP and anthropometric measurements of the children were taken at baseline, midpoint, and end point. ANOVA and descriptive statistics were used to analyzed data.

**Results:**

At baseline and end point, maternal KAP and the dietary diversity score of the children (mean age at end point 18.8 ± 2.9 mo) were assessed. Intervention mothers’ KAP improved (*p* < 0.001) at midpoint and end point compared to that of the control group, as did frequency of pulse consumption and Dietary Diversity Score (DDS) among children. At 9 months, the prevalence of stunting, wasting, and underweight was significantly reduced in the intervention group compared to the control group (*p* = 0.001).

**Conclusions:**

NE delivered by HEWs improved KAP of mothers regarding pulse consumption and dietary diversity of children led to improved nutritional status of the children. Training HEWs on the use of pulses for complementary food may be an effective way to improve the health of children in Ethiopian communities.

**Trial registration:**

Clinicaltrials.gov #NCT02638571.

Date of registration: 12/18/2015.

Prospectively registered.

## Background

Undernutrition in the first 1000 days is responsible for the annual death worldwide of over 3 million children under 5 years of age, and poor complementary feeding is one reason for undernutrition [1]. In Ethiopia, the prevalence of underweight, stunting, and wasting in children under 5 years of age is 24, 38, and 10% respectively [2]. Similar rates are found in the Southern Nations and Nationalities People Region (SNNPR) [2], the least urbanized region of the country (90% rural), where almost one in four live under the poverty line [3].

Exclusive breastfeeding is recommended for the first 6 months of a child’s life, followed by the introduction of complementary foods with continued breast-feeding. If complementary foods are insufficient in quality and/or quantity, nutritional deficiencies are at high risk of developing during the second half of infancy [4]. In Ethiopia, most complementary foods are made from unfortified cereal-based gruels [2, 5, 6], which are low in energy and nutrient density, leading to inadequacies of many nutrients including protein, iron, and zinc [7]. Indeed, in a 2016 Ethiopian survey, only 14% of children aged 6–23 months were found to consumed food from four or more food groups, and only 45% were fed the minimum acceptable diet [2]. In SNNPR, these rates were 12.5 and 41.9%, respectively [2].

In 2004, the Government of Ethiopia introduced the Health Extension Program to improve primary health services. Four years later, the National Nutrition Program (NNP) was introduced to address the growing concern of malnutrition among children under 5 years of age was introduced. A second NNP launched in 2016 emphasized the involvement of Health Extension Workers (HEWs) in the implementation of the program. HEWs are female high school graduates from the local community who speak the local language. As part of their job description, HEWs are mandated to provide nutrition education for mothers about essential child feeding practices [8, 9].

Pulses are important crops, providing high quality protein when blended with cereal [10]. They are also good sources of iron, zinc, and other important micronutrients when processed appropriately, which also improves protein digestibility [11, 12]. However, in Ethiopia, only 26% of children aged 6–24 months consume pulses. In SNNPR, although pulse crops are locally grown and available, only 9% of the population consumes pulses [13]. One of the major problems encountered in addressing malnutrition is lack of knowledge among mothers, family members, and health workers about the benefits of pulses for young children [4]. Previous pilot studies have shown that nutrition education on pulses improved the knowledge, attitude, and practice (KAP) of mothers in child feeding, and that nutrient intake and some parameters of growth improved [14, 15]. In these pilot studies, mothers received nutrition education via student researcher for a short interventional period. As an example, Mulualem et al. [14] provided 6 months of nutrition education to 80 mothers (compared to 80 controls) to improve mothers’ KAP and children’s nutritional status. Results of this small study indicated that the pulse messages improved KAP in mothers and weight gain in children, but no significant change in stunting [14]. Sustainability of a researcher-led program is uncertain, and 6 months of intervention may not have been sufficient to have a sustainable impact on improved KAP in rural mothers. Thus, little is known about the effectiveness and sustainability of a nutrition education program conducted through the regular activities of the HEWs. The main objective of the study was to assess the effectiveness of nutrition education on pulse-incorporated complementary food to the wider rural community through the government health system to improve maternal KAP, dietary diversity, and the nutritional status of children (6 to 24 months). The hypothesis was that nutrition education would improve mothers’ KAP of pulse-incorporated complementary food and subsequently would improve nutritional status, and dietary diversity of their young children.

## Methods

### Study setting and participants

**Fig. 1:**
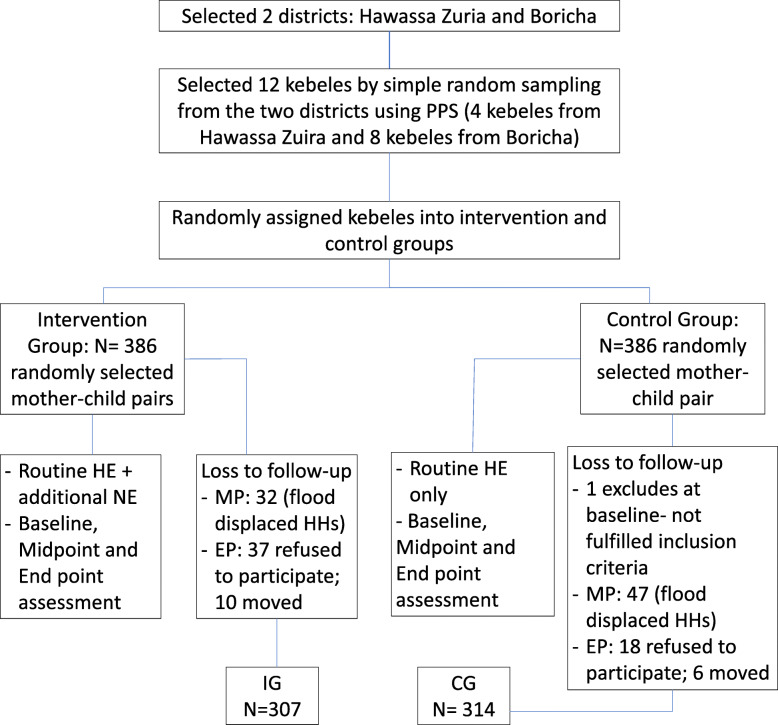
Flow Chart of the Study Design. (PPS: Proportion to Population Size; MP: Midpoint; EP: End Point; HE: Health education; NE: Nutrition Education)

This study was registered as Clinicaltrials.gov #NCT02638571, and the protocol, along with baseline results, have been published [16]. Briefly, this cluster-randomized intervention trial for community-based nutrition education was done in 12 kebeles/villages, selected from two districts of the Sidama Zone, Southern Ethiopia. Kebeles were randomly assigned to the intervention and control groups after stratification by districts using the lottery method, as the prevalence of child malnutrition and number of children was different in each district. A total of 772 mothers with children aged 6–15 months were recruited initially at the baseline. The total number of participants at baseline was 771 as one child was excluded due to not fulfilling the inclusion criteria. At the midpoint of data collection, the total number was 692 (354 in the intervention group and 338 in the control group), and at end point it was 621 (307 in the intervention group and 314 in the control group (Fig. 1). Mothers who have apparently healthy breastfeeding infants aged 6–15 months who were permanent residents in the area included in the study. Children who were receiving supplemental or those that were severely or moderately malnourished and had started therapeutic food were excluded from the study. Children who started therapeutic feeding excluded because their weight gain or improved situation would not show the effect of the intervention. The study was not blinded, because the districts were far apart (it was not possible to walk between kebeles and back in 1 day), did not share markets, health centers, and health posts, and study personnel did not overlap between areas.

### The intervention and education materials

Key messages were developed based on the Theory of Planned Behaviour (TPB) and Health Belief Model (HBM) principles [17, 18]. Health Extension Workers, two of whom were located in each kebele, were provided with 9 months of additional nutrition education, along with the usual health education. The HEWs provided the mothers in the intervention group with five main lessons. See the main lessons covered in intervention (Additional file 1: Table S1). An intervention with recipe demonstrations on preparation of porridge for complementary feeding using germinated pulse and cereal was given once a month and repeated again after midpoint (4.5 month) data collection. In addition, participating mothers in the intervention group were counseled by HEWs during house-to-house visits. The additional messages delivered to the intervention group were not included in the usual health education delivered to the control group. The control group received the usual health education provided in the area, which is mainly based on the essential nutrition action messages.

All HEWs are trained for 1 year before deploying for their services in their local community. They trained on Family Health as one of the training packages where a general nutrition education covered. For this study, a Training of Trainer (TOT) manual was used to provide additional training on pulses to HEWs in the treatment kebeles but not in control kebeles. This manual was developed and used by the Canadian International Food Security Research Fund (CIFSRF) for the “Scaling-up Pulse Innovations for Food and Nutrition Security” project [19]. Key messages included in the TOT manual were the importance of consuming food from all food groups and dietary diversification; the benefits of pulses; household pulse processing and preparation techniques, and the need to prepare and cook a variety of pulse-based dishes, including pulse-cereal mix complementary food. HEWs were trained for 3 days with demonstrations. At the same time, HEWs in the intervention group had refresher training in communication and counseling skills. In addition, HEWs were trained to use a quick guide when counseling mothers during house-to-house visits [20]. In the control sites, HEWs continued to provide routine health education. These HEWs had not been specially trained in using pulses in complementary food.

Before the intervention was introduced, the training material and counseling poster were pre-tested on purposively selected mothers to assess whether the content and format were realistic, understandable, culturally appropriate, visually appealing, and motivating. These mothers from the Hawassa Zuria district, who did not participate in the actual study, were provided with a half-day education and their understanding of the messages was assessed through discussion. Each picture on the poster was also assessed for its cultural acceptance.

The KAP of mothers regarding pulse consumption and feeding practices were collected at the baseline, midpoint, and end point of the intervention period. A standardized questionnaire was used to assess the mothers’ intentions to use cereal-pulse incorporated complementary food. Theory of Planned Behavior [18] and the Health Belief Model (HBM) was used to frame questions to assess the KAP of mothers based on the guidelines of Macias and Glasauer [17].

### Dietary diversity and growth assessment

Using a structured questionnaire, the mothers were asked about the type and number of meals consumed by their young children in the previous 24 h [21]. In addition, the Dietary Diversity Score (DDS) for each child was calculated based on the World Health Organizations (WHO) guidelines for measuring individual dietary diversity scores, using the following food groups to calculate the DDS: 1) grains, roots, and tubers; 2) legumes and nuts; 3) dairy products (milk, yogurt, cheese); 4) flesh foods (meat, fish, poultry, and liver/organ meats); 5) eggs; 6) vitamin-A rich fruits and vegetables; and 7) other fruits and vegetables [22]. The response of mothers was recorded as “Yes” if they said the child ate the particular food and “No” if they said the child did not eat the food. The answer “Yes” was recorded as 1 and “No” recorded as 0 and a sum of the total number of food groups consumed was calculated. The mothers also asked to estimate the amount of the food the child eats using locally used equipment for each child and the proportion of children consuming four or more food groups per day was determined. In addition to the number of meals, the frequency of the children’s pulse consumption was assessed using a frequency questionnaire to evaluate monthly consumption of pulses.

The anthropometry of the children was taken at baseline, midpoint, and end point using standardized techniques [23]. In brief, weight was measured using an electronic scale (Seca 770), and the children were draped in a light cloth of known weight during the measurement. The recumbent length was measured to the nearest 0.1 cm using the Shorr measuring board. The Middle Upper Arm Circumference (MUAC) of the left arm of young children was measured using arm circumference insertion tape. All anthropometric measurements were entered and analyzed using WHOAnthro version 3.2.2.

### Assessment of socio-demographic characteristics

Data on the socio-demographic characteristics of the participants, including those of the participants’ household, such as age, gender, ethnicity, income, and KAP of mothers, were assessed using a standard questionnaire adopted from previous studies [14, 24] with modifications. To assess the food insecurity of the households in the study area, a standardized questionnaire adapted from Food and Nutrition Technical Assistance (FANTA), the “Household Food Insecurity Access Scale,” was used [25]. As suggested by Ballard et al., 2011 [26], only the last three questions of the nine included to analyze food insecurity. These questions have been validated in low-income countries to measure household hunger. These three questions comprise the Household Hunger Scale (HHS). Food insecurity was assessed with a recall period of the last 4 weeks (30 days) prior to the data collection.

Household wealth status was measured by an asset-based (non-monetary) wealth index adopted from CSA [2]. During data collection, each participating household reported assets owned and other housing and sanitation-related characteristics. These included ownership of a radio, TV, mobile phone, TV, and bicycle, access to electricity, and quantity of livestock, land size, and level of income. Housing characteristics used in the wealth index calculation include the dwelling’s structure, number of rooms and bedrooms, and ownership (whether it is privately owned or rented). Each household received a score of 1 or 0 depending on whether it had the particular asset (1 = yes and 0 = No). Each binary variable was then weighted by the inverse of the proportion of households that owned the particular item or had the particular characteristics [27].

### Haricot beans for women’s empowerment in household decision making

Researchers associated with the larger project funded by CIFSRF attended an intervention nutrition education and demonstration session just prior to the midpoint data collection where mothers explained that although they understood the benefits of feeding their children pulses, they could not fully provide pulses as complementary food to their young children due to a shortage. At this time (late May and early June 2016), much of the population were affected by flooding that occurred due to an extended drought in the area. These climatic changes had prevented planting and/or reaping of pulses during the first harvest. The researchers decided, after the midpoint data collection to provide each of the mothers in the intervention group with a single gift of a two kg bag of quality haricot bean seed and a two kg bag of fertilizer to plant during the June–July planting season. Both intervention and control groups received pulse seeds and fertilizer for future planting from the study, with controls receiving seeds after the end point.

The women agreed to plant the seeds after a training session. Agriculture experts from Hawassa University’s College of Agriculture (partner institution) trained 386 mothers for 1 day on techniques of planting, applying fertilizer, and weeding. The mothers in the control group were later provided with one kg of haricot bean seed at the end of end point data collection. The provision of a small amount of a new variety haricot bean seed was meant to enable smallholder female farmers to improve their wellbeing and that of their families.

### Data analysis

Data were entered into SPSS version 20 software. Chi square and repeated measures Analysis of Variance (ANOVA) were used to investigate relationships between the pre- and post-intervention data on KAP of mothers, and growth and DDS of their children. ANOVA was used to compare means between the control and intervention groups, and when ANOVA was statistically significant, a post hoc test (Tukey HSD test) was used to determine the level of significance of values between and within groups. A value of *p* < 0.05 was considered as statistically significant.

## Results

### Study participants

The mean ± SD age of the mother was 25.5 ± 4.7 years: the majority (71%) were 21–30 years. About half the women were in primary school or had completed primary school. The mean ± SD age of young children was 9 ± 2.6 months, with about half of them between 6 and 8 months. A summary of the study participants’ socio-demographic characteristics is presented in Table 1.

**Table 1:** Socio-Demographic Characteristics of Study Participants at Baseline, Comparison of the Intervention Group (IG) and Control Group (CG), Southern Ethiopia, 2016 (*n* = 771)

Socio-demographic characteristics	IG n (%)	CG n (%)	***P***
Age of the mother (years)			
< 24	146 (37.8)	175 (45.5)	0.59
25–34	227 (58.8)	207 (53.8)	
> 35	13 (3.4)	3 (0.8)	
Marital status			
Married	365 (94.6)	379 (98.4)	**0.004**
Divorced	14 (3.6)	4 (1.0)	
Widowed	7 (1.8)	2 (0.5)	
Educational status			
Illiterate	150 (38.9)	147 (38.2)	0.58
Read and write	236 (61.2)	238 (61.8)	
In charge of food purchase			
Yes	150 (38.9)	158 (59.0)	0.54
No	236 (61.1)	158 (41.0)	
Source of income generating activities by woman			
Yes (petty trade, day labor)	62 (16.1)	49 (12.7)	0.19
No	324 (83.9)	336 (87.3)	
Household size			
Low (1–4 family members)	97 (25.1)	124 (32.2)	
Medium (5–10 family members)	222 (57.5)	190 (49.4)	0.45
Large (> 10 family members)	67 (17.4)	71 (18.4)	
Age of the children (months)			
6–8	172 (44.6)	197 (51.2)	0.19
9–11	123 (31.9)	110 (28.6)	
12–15	91 (23.6)	78 (20.3)	
Sex of children			
Male	211 (54.7)	201 (52.2)	0.50
Female	175 (45.3)	184 (47.8)	
Wealth Index			
Poor	255 (66.1)	229 (59.5)	0.55
Better	131 (33.9)	156 (40.5)	
Household hunger			
No household hunger	77 (19.9)	140 (36.4)	0.10
Mild household hunger	46 (11.9)	27 (7.0)	
Moderate household hunger	125 (32.4)	84 (21.8)	
Severe household hunger	138 (35.8)	134 (34.8)	

*P* is significant at < 0.05

### Knowledge, attitude, and practices of mothers

**Fig. 2:**
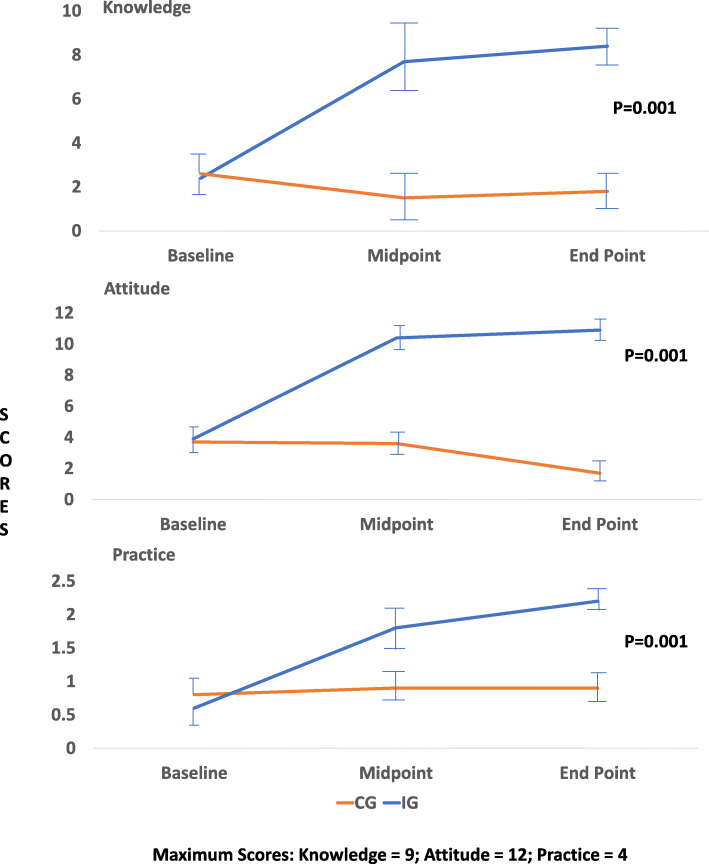
Mean Knowledge, Attitude, and Practice of Mothers about Pulse-Incorporated Complementary Foods, Sidama Zone, 2016 (*N* = 621)

Both the intervention and control group mothers had low scores on KAP at baseline. At midpoint, KAP improved in the intervention group (Fig. 2). After the 9-month nutrition education, mothers’ mean knowledge (*p* = 0.001) and practices (*p* = 0.001) significantly improved, but the attitude score remained the same as the midpoint score. There was a significant main effect of nutrition education on knowledge (*F* = 488.498; df = 2; *p* = 0.001), attitude (*F* = 375.221; df = 2; *p* = 0.001), and practices (*F* = 201.431; df = 2; *p* = 0.001) within groups. A similar significant effect was seen between groups on knowledge (*F* = 3071.99; df = 1; *p* = 0.001), attitude (*F* = 1297.50; df = 1; *p* = 0.001) and, practices (*F* = 158.98; df = 1; *p* = 0.001).

### Household processing of pulses and preparation of complementary foods by the mothers

At baseline, none of the mothers from either the intervention or control group indicated having ever soaked and germinated pulse products. At midpoint, 132 (43%) from the intervention and 29 (9.2%) from the control group reported that they had soaked and germinated pulses. Only 13 (4.2%) from the intervention group could recall from memory all the steps in household processing techniques (sorting, soaking, draining and soaking for 48 h, sun drying, roasting in a warm pan, and milling), and no members of the control group could describe all of these steps. In addition, only five women (1.6%) from the intervention and three (1%) from the control group were able to recall the right proportion of cereal-pulse mix, which is 3/4th cereal and 1/4th pulse. At end point, 214 (69.7%) from the intervention and 37 (11.8%) from the control group reported that they soak and germinate pulse crops; only 23 (7.5%) from the intervention group could recall from memory all six steps of the household processing techniques, while none of the control group could. One hundred and forty-six (47.6%) from the intervention and 15 (4.8%) from the control group had learned the right proportion of cereal-pulse mix for preparing complementary food (Table 2).

**Table 2:** Percentage of Selected Practice Variables of Intervention Group (IG) and Control Group (CG), Southern Ethiopia, 2016

Variables	Midpoint		***X***^**2**^	End point		***X***^**2**^
	N	%		N	%	
Prepare pulse incorporating CF						
IG	289	94	**0.001**	302	98.4	**0.001**
CG	211	67.2		213	67.7	
Soak and germinate pulse crops						
IG	132	43.0	**0.001**	414	69.7	**0.001**
CG	29	9.2		37	11.8	
Show 3 or more steps of household processing techniques						
IG	88	28.6	**0.001**	117	38	**0.001**
CG	1	0.3		3	0.9	
Mix cereal and pulse crops in the right proportions						
IG	5	1.6	**0.001**	146	47.6	**0.001**
CG	3	1.0		15	4.8	

Midpoint: 4.5 months; End point: 9.0 months. IG *n* = 307; CG *n* = 314

### Pulse consumption, dietary diversity, meal frequency, and nutrient intake

**Fig. 3:**
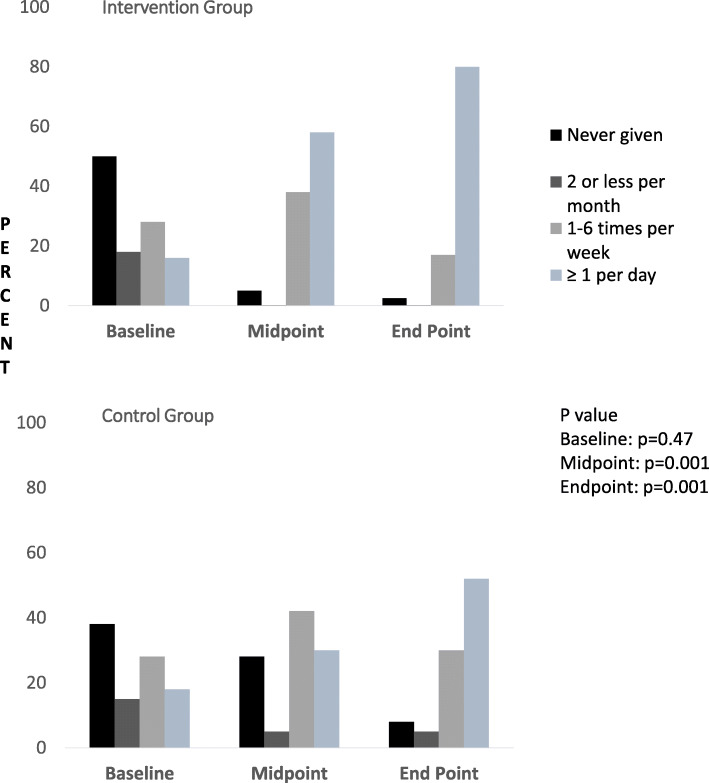
Frequency of Pulse Consumption by Children, Sidama Zone, 2016 (Intervention group, IG: *N* = 307; Control Group, CG: *N* = 314)

Haricot bean was the most commonly used pulse crop in the study area. Mothers who reported using pulses once or more than once per day in complementary food increased from 11.1% at baseline to 80.1% at end point in the intervention group and from 15.3% at baseline to 58.6% at end point in the control group. At baseline, there was no significant difference between the intervention and control groups in children’s consumption of pulses (*p* = 0.47). At midpoint and end point, there was a significant difference in consumption of pulses between the control and intervention groups (*p* = 0.001). Figure 3 shows the frequency of pulse consumption by young children in both the intervention and control groups.

Mean dietary diversity at baseline was similar for the intervention children (2.1 ± 1.0) and controls (2.2 ± 0.8); at midpoint, values were 2.5 ± 0.8 and 2.2 ± 0.9, respectively and, at end point, 3.7 ± 1.4 and 3.2 ± 1.4, respectively. At baseline, only 23 (7.5%) from the intervention and 12 (3.8%) from the control group had consumed food from four or more food groups in the last 24 h prior to data collection. At midpoint, 27 (8.8%) from the intervention and 26 (8.3%) from the control group had consumed food from four or more food groups in the same period. At end point, 158 (51.5%) from the intervention and 136 (43.3%) from the control had consumed food from four or more food groups. There was a statistically significant difference between baseline and midpoint diet diversity (*p* = 0.001) and between midpoint and end point (*p* = 0.001) between and within groups, which indicated change overtime in both groups. Table 3 summarizes the food groups consumed by young children in the 24 h prior to data collection at baseline, midpoint, and end point.

**Table 3:** Food Groups of Young Children in the last 24 Hours Prior to Data Collection Date at Baseline, Midpoint and End point, Southern Ethiopia, 2016

Food group	Baseline N (%)		Midpoint N (%)		End point N (%)	
	IG	CG	IG	CG	IG	CG
	Yes	Yes	Yes	Yes	Yes	Yes
Cereal & root crops	236 (76.9)	273 (86.9)	281 (91.5)	281 (89.5)	304 (99)	312 (99.4)
Pulse & nuts	96 (31.3)	121 (38.5)	189 (61.1)	135 (43)	264 (86)	81 (25.8)
Milk & milk group	27 (8.8)	12 (3.8)	31 (10.1)	10 (3.2)	134 (43.6)	128 (40.8)
Meat & organ meat	0	0	0	0	4 (1.3)	6 (1.9)
Egg	27 (8.8)	12 (3.8)	42 (13.7)	64 (20.4)	147 (47.9)	168 (53.5)
Vitamin A rich fruits and vegetables	0	0	105 (34.2)	74 (23.6)	192 (62.5)	198 (63.1)
Other fruits and vegetables	265 (86.3)	283 (90.1)	116 (37.8)	133 (42.4)	106 (34.5)	99 (31.5)
**Mean Diet Diversity Score**	**2.1 ± 1.0**	**2.2 ± 0.8**	**2.5 ± 0.8**	**2.2 ± 0.9**	**3.7 ± 1.4**	**3.2 ± 1.4**

*IG* Intervention Group (*n* = 307), *CG* Control Group (*n* = 314)

### Nutritional status of children

Anthropometric measurements were measured in the study children at baseline, midpoint, and end point. At baseline, there was no significant difference between the intervention and control groups in all anthropometry measurements and anthropometry indices. At end point, the mean age of male children was 18.8 ± 2.9 mo and female children was18.7 ± 2.9 mo. After intervention there was a significant difference (*p* < 0.05) in all anthropometric indices in the intervention group compared to the control group, as well as differences over time (Table 4). At baseline, low height and weight measurements were reflected in the high prevalence of stunting, wasting, and underweight. At midpoint and end point measurements, there were increases in the prevalence of stunting in both intervention and control groups; however, wasting and underweight improved only in the intervention group.

**Table 4:** Anthropometric Status of Children at Baseline, Midpoint and End point at Sidama Zone, Southern Ethiopia, 2016

Anthropometry measurements	Mean (SD) (***N*** = 771)	Mean (SD) (***N*** = 621)	
	Baseline	Midpoint	End point
Weight (kg)			
Intervention	8.3 (±1.3)^a^	9.3^b,x^	10.2^c,x^
Control	7.3 (±1.2)^a^	8.7^b,y^	9.4^c,y^
Length (cm)			
Intervention	69.3 (± 4.7)^a^	74.3^b,x^	78.1^c,x^
Control	68.9 (±1.1)	73.6^b,x^	77.1^c,y^
MUAC (cm)			
Intervention	13. 6 (±1.1)^a^	13.8^b,x^	14.4^c,x^
Control	13.6 (±1.0)^a^	13.4^b,y^	13.9^c,y^
**Anthropometric indices**			
Wasting (weight-for-length z score)			
Intervention	−0.44 (±1.2)^a^	0.06^b,x^	0.28^c,x^
Control	−0.73 (±1.1)^a^	−0.5^b,y^	−0.43^c,y^
Stunting (length-for-age z score)			
Intervention	−1.05 (±1.4)^a^	−1.2^b,x^	−1.5^c,x^
Control	−1.10 (±1.2)^a^	1.3^b.x^	−1.7^c,y^
Underweight (weight-for-age z score)			
Intervention	0.23 (±1.2)^a^	−0.5^b,x^	− 0.5^c,x^
Control	−0.13 (±1.1)^a^	−1.3^b,y^	− 1.2^c,y^
MUAC z score			
Intervention	−0.68 (±1.0)^a^	−0.7^b,x^	− 0.4^c,x^
Control	−0.61 (±1.0)^a^	−1.0^b,y^	− 0.7^c,y^

*SD* Standard Deviation; Values in rows are significantly different (*p* < 0.05) from the baseline if they have the letter “b” or “c”; values with the letter “c” are significantly different from values having “b.” Values for intervention and control in the midpoint and end point columns are significantly different if they have the letter “x” or “y”; values with the letter “x” are significantly different from values of “y” (*p* < 0.05). MUAC: mid-upper arm circumference. IG = Intervention Group (*n* = 307); *CG* Control group (*n* = 314)

Differences between the intervention and control groups were largely seen in all three measures: stunting, wasting, and underweight during the course of the study. At end point, the prevalence of stunting increased more in the control group than in the intervention group, and the difference was significant (*p* = 0.02). At baseline, 14.4% in the intervention group and 25.8% in the control group were wasted, and prevalence did not change much at midpoint (15.3 and 28.1%, respectively); at end point, the prevalence of wasting had significantly increased (*p* = 0.001) in the control group (29.7%) over time, while the intervention group (11.8%) had declined from baseline, showing a significant positive effect of the intervention. At baseline, 32.9% of children in the intervention group and 34% of children in the control group were underweight; at midpoint only the control group showed a worsening (47.4% of children). Both groups had a decreased prevalence of underweight (11.7 and 29.3%, respectively) at end point, but more children were underweight in the control group (*p* = 0.001). These changes in the children in the two groups were reflected in MUAC measurements. At baseline, 35% of children from the intervention group and 37% of children from the control group had lower MUACZ-scores. Although the number of children who had lower MUACZ scores increased in both the intervention (40%) and control group (54%), at midpoint, more children in the control group had lower MUACZ-scores compared to the intervention group. At end point, the number of children with low MUACZ scores had decreased in both the intervention (28%) and control (38.6%) groups compared to midpoint. However, at end point the number of children who had low MUACZ-scores was greater in the control group than in the intervention group (*p* = 0.001) (Table 4).

### Women’s empowerment in household decision making

All participating mothers (*n* = 386) in the intervention group reported that they had planted the haricot bean seed provided to them after the midpoint data collection. The seeds were later harvested following the end point data collection. Several visits to the field were carried out by the agriculture agents and the project team to a selected group of women; however, we did not collect information on how much they harvested, how much they kept for consumption, or how much they sold during end point data collection.

## Discussion

The first 2 years of life are critical to reducing problems related to malnutrition [28]. The current study scaled-up this strategy by implementing a community-based nutrition education intervention entirely delivered through HEWs. The results of the study showed that mothers’ KAP regarding pulse-incorporated complementary food was low at baseline in both the intervention and control groups. After 4 months of education (i.e., at midpoint), mothers in the intervention group improved KAP, which continued through the 9-month period. Almost all mothers in the intervention group had good knowledge about the benefits of pulses, household food processing techniques, and methods of preparation; they also started preparing complementary food using pulse crops. This current study findings are similar to other large intervention studies in other counties such as China [29, 30] and Kenya [31], which showed improved mothers’ knowledge about feeding practices when health service providers were used to provide nutrition education.

Improvements in the intervention group were seen in many measures. Frequency of pulse consumption significantly increased in the intervention group as most mothers started using pulses as a complementary food more than once per day. Frequency of pulse consumption improved in the intervention group after midpoint. Changes in knowledge and attitudes observed at the midpoint of the study were similar to the endpoint. However, the practice change was more significant at the end of the study than at midpoint. This finding may be attributed to the seed provision, which has motivated mothers to practice rather than nutrition education alone. Furthermore, there was a significant difference in mean dietary diversity between the intervention and control groups. At the end of the intervention, consumption of pulses and nuts group improved significantly in the intervention group. Although the mean diet diversity in both groups improved at the midpoint and end point, the intervention group had a slightly higher mean diet diversity score than the control group. However, at end point, both groups were preparing foods from fewer food groups per day than recommended (i.e., less than four) [22]. Meal frequency in young children was significantly higher in the intervention group at both midpoint and end point. This study results were similar to those of a Chinese study, which was designed to improve the feeding practices of young children and found improved diet diversity as well as meal frequency in the intervention group [30].

Stunting increased with age in both groups, but by end point, less children were stunted in the intervention group than in the control group, which could reflect the positive impact of the intervention. The study findings were consistent with those of similar studies in Peru and China. A Peruvian study found a significant difference in stunting between the intervention and control groups and a reduced rate of stunting in the intervention group at the end of the study [32]. A Chinese study found a marginal significant difference in stunting between treatment and comparison groups [29]. Other similar studies have also observed a decreased rate of stunting and improvement in linear growth at the end of intervention, although these studies found no statistically significant difference between the treatment and comparison groups [33–36]. However, other studies have found neither improvement nor significant differences in stunting between the treatment and comparison group [14, 31]. The range of the nutrition education period in most studies ranged from six to 18 months, a short time to find a significant change in stunting. In the current study, the prevalence of stunting increased with age, consistent with previous studies [33–36]. This increase in stunting with age could occur because of factors such as infectious disease, poor maternal malnutrition during pregnancy, poor hygiene, and poverty [31], all of which limit the impact of an intervention over a relatively short period. In the same way, in the current study, underweight also increased with age in both intervention and control groups. However, the prevalence of wasting decreased over time in the intervention group, whereas the number of children in the control group who were wasted increased at the end point.

In our study, we found household processing practices such as germination to be difficult tasks for mothers to adopt. The number of mothers who were using soaked and germinated pulse crops for complementary food was low. Only 43 and 69.7% of mothers in the intervention group reported soaking and germination pulses at midpoint and end point, respectively. However, the results showed that more mothers performed household processing techniques in the intervention group than in the control group. The number of mothers who adopted practices for soaking and germination of pulses in this study was higher than that in a similar study in Malawi, where only 25% of participants adopted the practices from the message [37]. This difference could be due to the length of intervention, which was shorter than this current intervention. In general, the effect of nutrition education on mothers’ knowledge and practice was high at both the midpoint and end point of this study, but, attitude did not change after the midpoint, possibly indicating that an intervention of approximately 5 months could be sufficient to change mothers’ attitude.

The strength of this study was the involvement of trained local HEWs who can speak the local language in delivering the nutrition messages. The intervention contributed to women’s empowerment by using HEWs and training mothers in their own communities. A peer mentoring approach can have positive impacts on the knowledge, confidence, and attitudes of participants [38]. We have described training the HEWs elsewhere [39]. In addition, multiple educational theories, namely Theory of Planned Behavior (TPB) and Health Belief Model (HBM) were used in designing the messages. TPB helped to identify factors that may influence pulse consumption in feeding practices through a combination of attitudes towards consumption of pulses, household processing techniques, cultural influences, and behavioral control that results in the formation of an intention [18]. HBM helped in addressing the problem behaviour, in this case the risk of inappropriate feeding practice and low consumption of pulses leading to undernutrition [17]. The limitations of the study included the potential for information contamination between intervention and control sites. Another limitation of the study was not assessing whether there were differences in the motivation of the HEWs between treatment and control kebeles, which could affect the outcome of the study. The distribution of seed after midpoint was an unexpected addition to the study; however, harvesting of these planted seeds had not occurred before end point, and thus this additional variable did not impact the results at 9 months.

## Conclusions

This study found that nutrition education delivered through HEWs was successful in improving young children’s consumption of pulses and their nutritional status. The pulse nutrition education given to mothers by HEWs for 9-month helped in improving the maternal KAP and frequency of pulse consumption as well as the nutritional status of their children. The study showed that training local HEWs is an effective way of promoting locally available nutritious foods and improving the health of local communities, particularly in low-income countries. Specifically, we showed soaking and germination and to incorporating germinated pulse product into complementary food were skills that needed to be effectively taught. The HEW’s network was important to bring changes in maternal KAP. It is necessary to strength the training of HEWs on germination particularly as the skills were not easily adopted in the communities. It is also showed that scaled-up the messages using established health education networks is possible at the national level. Finally, we recommend a future study on cost-benefit analysis of enhanced nutrition education that integrates pulse use into the HEWs information package and training.

## Supplementary information

**Additional file 1: Table S1.** Lesson Plan for Mothers’ Pulse Education. This is a content of nutrition education module covered during the intervention to teach mothers in the intervention group.

## Data Availability

The dataset generated and/or analyzed during the current study available from the corresponding author on reasonable request.

## Abbreviations

ANOVA: Analysis of variance
CIFSRF: Canadian International Food Security Research Fund
CSA: Central Statistics Agency
DDS: Dietary Diversity Score
FANTA: Food and Nutrition Technical Assistance
HBM: Health Belief Model
HEW: Health Extension Worker
HHS: Household Hunger Scale
KAP: Knowledge, Attitude, Practice
MUAC: Mid-Upper Arm Circumference
NNP: National Nutrition Program
SNNPR: Southern Nations and Nationalities People’s Region
TPB: Theory of Planned Behavior
TOT: Training of Trainers
WHO: World Health Organization
