# Supplementary material for: Scaled-up nutrition education on pulse-cereal complementary food practice in Ethiopia: a cluster-randomized trial
Source: BMC Public Health. 2020 Sep 22;20:1437. doi: 10.1186/s12889-020-09262-8 (PMC7507676; doi:10.1186/s12889-020-09262-8)
Supplement: Supplementary file 1 — Additional file 1: Table S1. Lesson Plan for Mothers’ Pulse Education. This is a content of nutrition education module covered during the intervention to teach mothers in the intervention group. [file 12889_2020_9262_MOESM1_ESM.docx]

Additional file 1: Table S1. Lesson Plan for Mothers’ Pulse Education

| **Session title** | **Contents** |
| --- | --- |
| Why young children need a well-balanced diet complementary food? | - Definition of balanced diet - Young children’s susceptibility to undernutrition (exposure to repeated infectious diseases, low physical and mental performance) - The need of balanced complementary food |
| Food groups and benefits of pulses | - Nutrient content and food groups - Enriching complementary foods using pulses - Proportion of cereal and pulses for preparation of commentary food - Recipe demonstration (preparation of porridge from germinated pulses and cereal for complementary feeding) |
| Household processing techniques | - Soaking - Germination - Drying and roasting - Milling - Safe storage of processed flour |
| Review | - In the final session, the materials used in the previous discussion were reviewed and summarized through discussion with mothers. |
